# Endothelial function following interval exercise plus low‐calorie diet treatment in obese females

**DOI:** 10.14814/phy2.14239

**Published:** 2019-09-25

**Authors:** Nicole M. Gilbertson, Stephanie L. Miller, Natalie Z.M. Eichner, Steven K. Malin

**Affiliations:** ^1^ Department of Kinesiology University of Virginia Charlottesville Virginia; ^2^ Division of Endocrinology & Metabolism Department of Medicine University of Virginia Charlottesville Virginia; ^3^ Robert M. Berne Cardiovascular Research Center University of Virginia Charlottesville Virginia

**Keywords:** Energy deficit, fitness, flow‐mediated dilation, insulin

## Abstract

We determined if interval exercise plus a low‐calorie diet (LCD + INT) increases endothelial function more than an energy‐matched LCD. Obese women (47.2 ± 2.6y, 37.5 ± 1.3kg/m^2^) were randomized to 13 days of a LCD (*n* = 12; mixed meals of ~ 1200kcal/d) or LCD + INT (*n* = 13; 12 supervised 60‐min INT bouts of 3 min at 90% and 50% HR_peak_). LCD + INT subjects received 350kcal postexercise to equate energy availability with LCD. Fitness (VO_2_peak) and body composition (BodPod) were determined and a 120 min, 75 g oral glucose tolerance test was performed to examine fasting and postprandial flow‐mediated dilation (FMD, endothelial function), respiratory exchange ratio (RER) via indirect calorimetry as well as glucose and insulin incremental area under the curve (iAUC_120min_). LCD + INT increased VO_2_peak (*P* = 0.02) compared with LCD, and both treatments decreased fat mass (*P* < 0.001) and insulin iAUC_120min_ (*P* = 0.03). There was no overall treatment effect on fasting or iAUC_120min_ FMD. However, in participants who increased fasting endothelial function after each treatment (Δ > 50%; LCD *n* = 5, LCD + INT *n* = 7), LCD + INT increased fasted (*P* = 0.005) and decreased iAUC_120min_ (*P* = 0.003) FMD compared with LCD. Enhanced fitness correlated with increased fasting FMD (*r* = 0.43, *P* = 0.03) and diminished FMD iAUC_120min_ (*r* = −0.44, *P* = 0.03). Decreased FMD iAUC_120min_ correlated with reduced glucose iAUC_120min_ (*r* = 0.64, *P* = 0.001) as well as increased 60‐min RER (*r *= −0.42, *P* = 0.04). Low baseline fasting and iAUC_120min_ FMD was also linked to enhanced fasting and iAUC_120min_ FMD post‐treatment (*r* = −0.71, *P* < 0.001; *r *= −0.89, *P* < 0.001, respectively). In conclusion, increasing fitness via INT may increase the effect of LCD on lowering cardiovascular disease risk in obese women.

## Introduction

Endothelial dysfunction is a subclinical link to cardiovascular disease (CVD) in obese adults (Lobato et al., 2012; Ortega et al., 2016). Obesity is related to endothelial dysfunction through, in part, structural changes of the intima and media of the vascular wall as well as decreased vasodilation signaled by nitric oxide and/or insulin that reduce nutrient delivery to metabolically active tissues (Steinberg et al., 1996; Arcaro et al., 1999; Jongh, 2004; Lobato et al., 2012). Recent reports suggested that females have higher endothelial dysfunction than males when matched for body mass index (BMI) (Suboc et al., 2013). Given that the absolute number of females living with and dying of CVD exceeds that of males (Roger et al., 2011), elucidating the optimal treatment to rescue endothelial dysfunction is needed in females.

Lifestyle therapy including a low‐calorie diet (LCD) (Joris et al., 2015) and/or aerobic exercise (Joris et al., 2015; Son et al., 2017) improves fasting endothelial function in both obese males and females. However, most lifestyle interventions ranging from 2–16 weeks improved fasting endothelial function as assessed by flow‐mediated dilation (FMD) are paralleled by >5% weight loss (Ziccardi et al., 2002; Cotie et al., 2014). This confounds the ability to determine whether it is caloric restriction and/or the rise in fitness from exercise that drives FMD. Interestingly, there is noted variation in FMD responses to lifestyle treatment (Green et al., 2014) that may be partially related to females having blunted FMD responses to continuous aerobic exercise (Pierce et al., 2011). In fact, 10 consecutive days of continuous high‐intensity exercise has either increased endothelial function in normal weight, older adults (Landers‐Ramos et al., 2016) or has had no effect in obese, middle‐aged adults with and without metabolic syndrome (Baynard et al., 2009). The reason for such discrepancy between short‐term studies is unclear, but it might suggest either an energy deficit above and beyond that of exercise alone or higher intensity of exercise is needed to raise FMD in obese females. While long‐term training studies demonstrate that high‐intensity interval (INT) exercise enhances fasting endothelial function with (Tjonna et al., 2008) or without weight loss (Tjonna et al., 2008; Ramos et al., 2015; Sawyer et al., 2016), only one study to date of low volume INT exercise (i.e., 3d/wk) for 2 weeks without weight loss demonstrates no impact on FMD in normal weight postmenopausal females (Klonizakis et al., 2014). It remains unclear if increasing the frequency of INT exercise sessions during a short‐term training intervention could increase FMD prior to clincally meaningful weight loss. To this extent, little is known regarding lifestyle therapy on postprandial endothelial function (Ramirez‐Velez et al., 2018). This is clinically relevant as postprandial hyperglycemia (Xiang et al., 2008; Lavi et al., 2009; Suzuki et al., 2012) and lipemia ( Ramirez‐Velez et al., 2018) are stronger predictors of CVD risk than fasting alone (Lin et al., 2009; Ansar et al., 2011). Indeed, we previously showed that a single bout of high intensity exercise reduced circulating glucose in relation to increased endothelial function during a 75 g oral glucose tolerance test in obese adults with prediabetes who had low endothelial function (Malin et al., 2016). Whether INT exercise adds to the effects of LCD on fasted and postprandial endothelial function compared to an energy deficit matched LCD alone in obese females is unknown. Thus, we tested the hypothesis that LCD + INT would increase fasted and postprandial FMD more than LCD alone in obese females, and the rise in FMD would correlate with glucose metabolism.

## Methods

### Participants

Obese females (age: 47.2 ± 2.6 years, BMI: 37.5 ± 1.3 kg/m^2^) were recruited via advertisements in the local community, and some data were previously reported (Francois et al., 2018; Gilbertson et al., 2019). All participants completed a health history questionnaire and screening. Females were excluded from participation if they were physically active (>60 min/wk of physical activity), pregnant or lactating, smoking, diagnosed with chronic disease (e.g., CVD or diabetes), or on medications known to influence endothelial function (e.g., beta‐blockers, ACE‐inhibitors, etc.) or glucose tolerance (e.g., metformin, GLP‐1 agonist, etc.). Females were included if taking statins (LCD *n* = 1, LCD + INT *n* = 1), contraceptives (LCD *n* = 2, LCD + INT *n* = 3), or hormone replacement therapy (LCD *n* = 1, LCD + INT *n* = 0) as well as taking medication for asthma (e.g., albuterol, Montelukast; LCD *n* = 1, LCD + INT *n* = 2), muscle spasms (e.g., flexeril; LCD *n* = 1, LCD + INT *n* = 0), migraines (e.g., amerge, fioricet, topiramate; LCD *n* = 0, LCD + INT *n* = 1), fibromyalgia (e.g., lyrica, Cymbalta; LCD *n* = 0, LCD + INT *n* = 2), arthritis (e.g., meloxicam, plaquenil, restasis; LCD *n* = 0, LCD + INT *n* = 1), anxiety (e.g., clonazepam; LCD *n* = 0, LCD + INT *n* = 1), or depression (e.g., trazodone; LCD *n* = 0, LCD + INT *n* = 1). Participants were randomized to 2 weeks of a LCD (*n* = 12) or LCD + INT (*n* = 13). All subjects provided written and verbal informed consent as approved by the University of Virginia Institutional Review Board.

### Body composition and aerobic fitness

Body weight was measured on a digital scale and height was measured with a stadiometer to assess body mass index (BMI). Fat mass and fat‐free mass (FFM) were measured using BodPod (Concord, CA). Waist circumference was measured 2 cm above the umbilicus using a plastic tape measure. VO_2_peak was determined using a continuous progressive exercise test on a cycle ergometer with indirect calorimetry (Carefusion, Vmax CART, Yorba Linda, CA). Volitional exhaustion, respiratory exchange ratio (RER) >1.1, and a cadence below 60 rpm were used as indicators of VO_2_peak.

### Oral Glucose Tolerance Test (OGTT)

Metabolic control for 48 h prior to testing was implemented as previously reported for this trial (Francois et al., 2018; Gilbertson et al., 2019). In brief, subjects were instructed to refrain from caffeine and alcohol consumption as well as strenuous activity 48 h prior to testing. Subjects were also instructed to refrain from taking any medications or dietary supplements 24 h prior to testing. The last exercise bout was performed approximately 24 h before postintervention testing. Participants were admitted to the Clinical Research Unit at about 8:00 a.m. following an overnight fast. An intravenous catheter was placed in an antecubital vein and blood was collected prior to and during a 120 min, 75 g oral glucose tolerance test (OGTT). Fasting plasma was collected to measure vascular cell adhesion molecule 1 (VCAM‐1) and intercellular adhesion molecule 1 (ICAM‐1) to assess vascular inflammation. Circulating glucose, free fatty acids (FFA), and insulin were also determined at fasting and every 30 min up to 120 min to assess insulin sensitivity and glucose tolerance via incremental area under the curve (iAUC_120min_). Indirect calorimetry with a ventilated hood was utilized to quantify non‐protein respiratory exchange ratio (RER) at 0, 60, and 120 min of the OGTT to assess fuel selection.

### Flow‐Mediated Dilation (FMD)

Endothelial function was assessed via FMD at the brachial artery using Epiq 7C Ultrasound Machine (Philips Medical Systems, Andover, MA) when participants were fasted as well as at 60 and 120 min during the OGTT. All brachial artery assessments were performed in a quiet room, with participants in the supine position, and on the left arm. The brachial artery was imaged with a 12–3 MHz range linear transducer approximately 5cm proximal to the antecubital crease using B‐mode ultrasound. A blood pressure cuff was placed around the forearm immediately distal to the olecranon process. Following baseline brachial artery diameter imaging, the blood pressure cuff was inflated manually to 200 mmHg for 5 min and then deflated. The diameter of the brachial artery was imaged every 10 sec postdeflation for 2 min to capture post‐ischemic peak diameter. All images were stored in Digital Imagining and Communication in Medicine (DICOM) format for later analyses. Brachial artery images were analyzed by a single investigator (SLM) blinded to the conditions using commercially available software (Brachial Analyzer for Research v.6, Medical Imagining Applications LLC, Coralville, IA), and the software uses an automated method for near and far wall border detection and vessel diameter measurement in brachial ultrasound image sequences. Arterial diameter was measured as the distance (mm) between the intima‐lumen interfaces of the anterior and posterior walls. FMD was defined as the relative change in postreactive hyperemia diameter compared to the baseline diameter as we performed before (Malin et al., 2016) and iAUC_120min_ was calculated to assess postprandial stimulation.

### Low‐Calorie Diet (LCD)

Prior to pre‐intervention testing, subjects were instructed to record their ad‐libitum dietary intake for 3 days. Participants then underwent a 13 day LCD (1000–1200 kcal/d) based on pre‐operative diets recommended to obese adults undergoing bariatric surgery. Meal replacement shakes were provided to participants for breakfast and lunch (Ensure^®^ Abbott Laboratories, USA, 8 fl. oz; providing 160 kcal, 16 g protein, 2 g fat, 19 g CHO), and participants were provided with a detailed menu with options for two 100 kcal snacks throughout the day and a sensible dinner that did not exceed 600 kcal (e.g., lean protein with vegetables). Empty shake containers were collected and 13 day food records were averaged throughout the intervention to assess compliance and caloric intake. Food intake was assessed using ESHA (Version 11.1, Salem, OR), and changes from pre‐ to post‐intervention are reported.

### Interval Exercise Training (INT)

Subjects randomized to LCD + INT completed 12 supervised INT sessions over a 13‐day period. Exercise duration at the desired intensity was progressively ramped up so that subjects completed 30 and 45 min of exercise on days 1 and 2, and thereafter, subjects completed 60 min of exercise per session with one rest day (i.e., day 7). Each exercise session began with subjects cycling for 3 min at 50% of heart rate peak (HRpeak) for a warm‐up and thereafter alternating 3‐min periods of cycling at 90% and 50% of HRpeak for a 60 min session. Subjects completed a light 5‐min cool‐down on the cycle at the end of each session to ensure an appropriate HR recovery. The study team conducted daily check‐ins with subjects to ensure participants were not experiencing excessive soreness or overuse injuries. A mixed‐meal shake (Ensure^®^ Abbott Laboratories, USA, 8 fl. oz; providing 350 kcal, 13 g protein, 11 g fat, 50 g CHO) was provided to participants after each exercise session for consumption in effort to equate energy availability between treatments. Replacement was based on a previously reported 340 kcal expenditure per 60 min INT session in overweight/obese adults (Karstoft et al., 2013), and exercise energy expenditure (EEE) was calculated for each exercise session from VO_2_‐HR regression analysis (Malin et al., 2013).

### Biochemical analysis

Plasma glucose was analyzed real time using the glucose oxidase method (Yellow Springs, OH). FFA as well as insulin were assessed via colorimetric assay (Wako Chemicals, Richmond VA) and ELISA (Millipore, Billerica MA), respectively. VCAM‐1 and ICAM‐1 were also determined using ELISA (R&D Systems, Minneapolis, MN).

### Statistical analysis

Based on prior INT exercise (delta 3.85%, SD 2.8%) (Sawyer et al., 2016) and caloric restriction combined with exercise (delta 2.9%, SD 2.2%) (Cotie et al., 2014) work on fasting FMD in obese adults, it was determined that 9 participants (80% power and alpha of 0.05) would be needed to determine statistical significance between LCD and LCD + INT. Data were analyzed using SPSS Version 24 (IBM Analytics, Armonk, NY). Twenty‐five females (*n* = 12 LCD, *n* = 13 LCD + INT) were included in analyses for all variables except for blood parameters (*n* = 12 LCD, *n* = 12 LCD + INT), as blood was not collected for one subject (*n* = 1 LCD + INT) post‐intervention due to IV difficulty. Non‐normally distributed data were log‐transformed for analysis. There were no baseline differences between groups for any variable as assessed by independent t‐tests. All variables were analyzed using a repeated measure analysis of variance (ANOVA). Because of prior reports on inter‐subject variability in response to lifestyle treatment (Green et al., 2014), the change in fasting FMD from pre‐intervention to post‐intervention was calculated. Subjects were then categorized based off of their response or change in fasting FMD as being in the lower (LCD *n* = 7, LCD + INT *n* = 6) or upper (LCD *n* = 5, LCD + INT *n* = 7) 50th percentile for fasting FMD. There were no baseline differences in any variable across these four groups. Change from pre‐ to post‐intervention was determined for FMD as well as other metabolic and dietary variables, and a univariate ANOVA with post hoc independent samples t‐test as a secondary analysis was run to determine the intervention effect. Pearson’s correlation was used to assess associations. Data are mean ± SEM, and significance was set at *P* ≤ 0.05.

## Results

### Intervention characteristics

Both treatments similarly decreased total caloric (LCD −859.5 ± 222.2 vs. LCD + INT −775.2 ± 175.5 kcal; *P* ≤ 0.001), carbohydrate (LCD −329.2 ± 127.7 vs. LCD + INT −277.4 ± 95.3 kcal; *P* = 0.001), fat (LCD −414.9 ± 71.2 vs. LCD + INT −437.6 ± 78.4 kcal; *P* ≤ 0.001), and protein (LCD −117.9 ± 35.0 vs. LCD + INT −76.0 ± 32.9 kcal; *P* = 0.001) intake. Sodium (LCD −1553.6 ± 425.8 vs. LCD + INT −1407.0 ± 237.4 mg; *P* ≤ 0.001) and total fiber (LCD −6.0 ± 1.8 vs. LCD + INT −7.7 ± 1.5 g; *P* < 0.001) decreased similarly with both treatments, but there was no treatment effect on soluble fiber (LCD −0.36 ± 0.30 vs. LCD + INT −0.10 ± 0.17 g; *P* = 0.19). There was no difference in dietary intake following treatment among the 50th percentile groups (data not shown). LCD + INT exercise session adherence was 98.1%. Average exercise energy expenditure for all sessions was 371.1 ± 15.5 kcal/session and participants averaged 74.2 ± 1.1% and 89.5 ± 0.5% of HRpeak for low‐ and high‐intensity intervals for all sessions, respectively.

### Body composition and fitness

LCD and LCD + INT decreased BMI by 2.5 ± 0.2% and 1.3 ± 0.3% (*P* ≤ 0.001), respectively, and the reduction was greater following LCD (*P* = 0.01). However, both treatments reduced fat mass similarly (LCD −4.1 ± 0.9 vs. LCD + INT −3.4 ± 0.7%; *P* ≤ 0.001) (Table 1). The interventions had no effect on FFM or waist circumference (Table 1 ***)***. LCD + INT increased absolute and relative VO_2_peak compared to a decrease with LCD (*P* ≤ 0.02; Table 1). There were overall no differences at baseline or in the change of demographics and body composition based on 50th percentile groups (data not shown). However, LCD + INT increased absolute (*P* = 0.01) and relative (*P* = 0.02) VO_2_peak compared to LCD in subjects in the upper 50^th^ percentile following treatment.

**Table 1:** Effect of LCD or LCD + INT on subject characteristics, blood substrates, and adhesion molecules

	LCD		LCD + INT		ANOVA (*P*‐value)	
	Pre	Change	Pre	Change	T	G x T
Age (years)	45.7 ± 3.5	–	48.5 ± 3.8	–	–	–
Body composition						
Weight (kg)	103.6 ± 4.8	−2.8 ± 0.2	103.9 ± 5.8	−1.6 ± 0.3	<0.001	0.01
BMI (kg/m^2^)	37.8 ± 1.6	−0.9 ± 0.1	37.3 ± 2.0	−0.5 ± 0.1	<0.001	0.01
Fat mass (kg)^a^	50.7 ± 4.0	−2.0 ± 0.4	50.1 ± 4.5	−1.6 ± 0.3	<0.001	0.55
Lean mass (kg)	50.1 ± 1.4	−0.5 ± 0.2	51.3 ± 2.5	0.1 ± 0.3	0.30	0.14
WC (cm)^a^	114.6 ± 3.7	0.5 ± 1.2	111.6 ± 4.7	−1.6 ± 1.1	0.49	0.23
Aerobic fitness						
VO_2_peak (L/min)	1.9 ± 0.1	−0.2 ± 0.1	1.9 ± 0.1	0.1 ± 0.1	0.58	0.009
VO_2_peak (mL/kg/min)^a^	19.0 ± 1.3	−0.6 ± 0.5	18.5 ± 1.4	1.3 ± 0.6	0.50	0.02
Fasting RER	0.84 ± 0.01	−0.05 ± 0.02	0.81 ± 0.02	−0.02 ± 0.01	0.002	0.29
60 min RER	0.90 ± 0.02	−0.10 ± 0.01	0.87 ± 0.02	−0.04 ± 0.02	<0.001	0.09
120 min RER	0.91 ± 0.01	−0.08 ± 0.02	0.89 ± 0.01	−0.01 ± 0.01	<0.001	0.004
Glucose (mg/dL)						
Fasting	96.9 ± 1.4	−3.6 ± 2.2	98.3 ± 2.2	−2.6 ± 1.9	0.03	0.88
120 min iAUC	2521.5 ± 846.1	704.3 ± 630.6	4642.6 ± 541.5	−158.2 ± 728.6	0.45	0.47
FFA (mEq/L)						
Fasting	0.50 ± 0.04	0.13 ± 0.06	0.56 ± 0.05	0.03 ± 0.04	0.03	0.13
120 min iAUC	−34.4 ± 2.9	−5.6 ± 5.5	−39.6 ± 3.8	4.1 ± 2.7	0.82	0.13
Insulin (μU/mL)						
Fasting	16.4 ± 2.5	−4.1 ± 1.9	20.6 ± 5.3	0.2 ± 4.6	0.43	0.39
120 min iAUC^a^	9436.4 ± 1231.4	−1516.6 ± 1032.1	12480.0 ± 2195.7	−2594.0 ± 1038.1	0.03	0.93
Inflammation						
ICAM‐1 (ng/mL)	217.8 ± 15.3	−25.7 ± 5.9	233.7 ± 31.0	−9.9 ± 8.0	0.002	0.13
VCAM‐1 (ng/mL)	581.5 ± 45.4	−22.5 ± 43.4	573.1 ± 29.8	−8.7 ± 28.4	0.55	0.79

Data are means ± SEM, and absolute change is reported. Body mass index (BMI); waist circumference (WC); respiratory exchange ratio (RER); incremental area under the curve (iAUC); free fatty acids (FFA); intercellular adhesion molecule 1 (ICAM‐1); vascular cell adhesion molecule 1 (VCAM‐1); Treatment Effect (T); Group x Treatment Interaction (GxT).

a Non‐normally distributed data are presented in raw version for ease of interpretation.

### Blood chemistries and fuel selection

Plasma VCAM‐1 did not change after either treatment, but both interventions lowered plasma ICAM‐1 (*P* = 0.002; Table 1). LCD and LCD + INT reduced fasting glucose (*P* = 0.04; Table 1) with no effect on glucose iAUC_120min_ (Table 1). Fasting insulin was not changed, whereas insulin iAUC_120min_ (*P* = 0.03; Table 1) was reduced comparably following LCD and LCD + INT, suggesting increased insulin sensitivity. Fasting FFAs (*P* = 0.03; Table 1) increased after LCD and LCD + INT but there was no effect on FFA iAUC_120min_ (Table 1). Both treatments decreased fasting RER (LCD −6.2 ± 2.2 vs. LCD + INT −2.6 ± 1.8%; *P* = 0.002). LCD and LCD + INT decreased postprandial RER (*P* ≤ 0.001), but the reduction was greater following LCD at 120 min (*P* = 0.004; Table 1). There was no difference at baseline or in the change in blood chemistries and fuel selection based on 50^th^ percentile groups following treatment (data not shown).

### FMD

**Figure 1:**
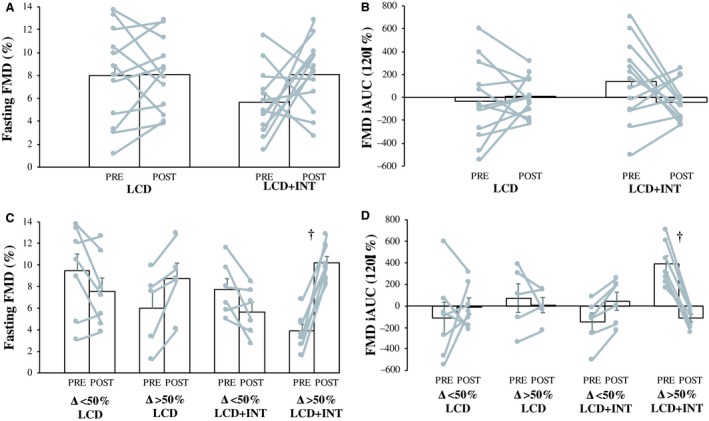
Effect of a low calorie diet (LCD) and low calorie diet plus interval exercise (LCD + INT) interventions on relative fasting (A) and 120 minute incremental area under the curve (iAUC_120min_) (B) flow mediated dilation (FMD) in all study participants. Effect of LCD and LCD + INT interventions on relative fasting (C) and iAUC_120min_ (D) FMD in study participants in the lower (Δ < 50%; LCD *n* = 7, LCD + INT *n* = 6) and upper (Δ> 50%; LCD n = 5, LCD + INT *n* = 7) 50th percentile. Bars are presented as mean ± standard error of the mean (SEM). Grey lines are individual data points pre‐intervention (PRE) to post‐intervention (POST). † Denotes a significant (*P* ≤ 0.005) difference from Δ > 50th% LCD.

There was no difference in artery dimeter before or after either treatment (Table 2). Moreover, LCD and LCD + INT had no effect on fasting or iAUC_120min_ FMD (*P ≥ *0.14; Fig. 1). When comparing subjects in the upper (LCD *n* = 5, LCD + INT *n* = 7) 50th percentile, LCD + INT increased fasted FMD (6.3 ± 0.8 vs. 2.8 ± 0.9%, *P* = 0.005) more than LCD, and LCD + INT decreased FMD iAUC_120min_ (−499.3 ± 91.6 vs. −64.6 ± 108.4%, *P* = 0.003) compared to LCD (Fig. 1). There was no difference at baseline or in the change in baseline artery diameter based on 50th percentile groups following treatment (data not shown).

**Table 2:** Effect of LCD or LCD + INT on pre‐occlusion brachial artery diameter

	LCD		LCD + INT		ANOVA (*P*‐value)	
	Pre	Change	Pre	Change	T	G x T
Fasting Diameter (mm)	3.55 ± 0.15	−0.07 ± 0.11	3.68 ± 0.12	−0.02 ± 0.05	0.43	0.71
60 min Diameter (mm)	3.64 ± 0.14	−0.06 ± 0.11	3.67 ± 0.12	−0.08 ± 0.04	0.22	0.81
120 min Diameter (mm)	3.88 ± 0.12	0.04 ± 0.10	3.93 ± 0.12	0.10 ± 0.07	0.24	0.63

Data are mean ± SEM, and absolute change is reported. Treatment Effect (T); Group x Treatment Interaction (G x T).

### Correlations

**Figure 2:**
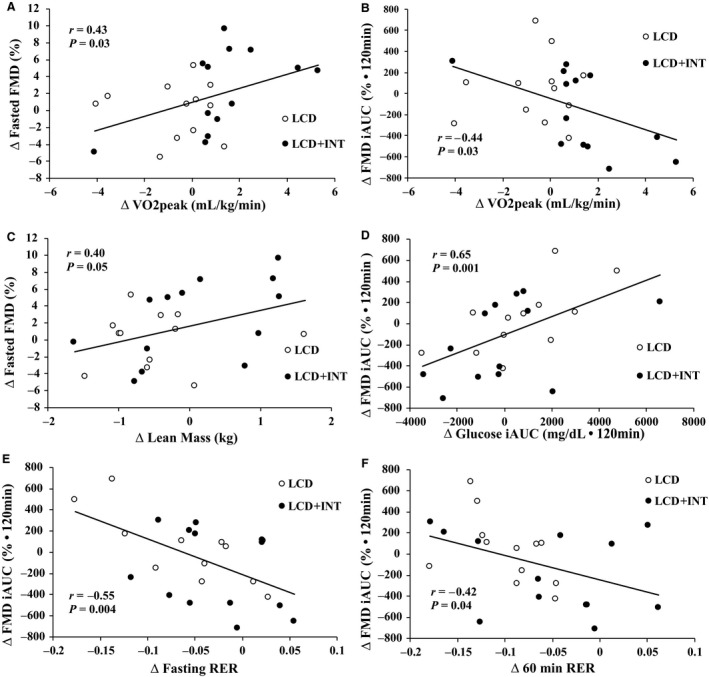
Correlations in flow mediated dilation (FMD) to cardiometabolic outcomes following the intervention. The change (Δ) in fitness to the Δ in fasting FMD (A) and Δ in FMD incremental area under the curve (iAUC_120min_) (B). The Δ in lean body mass to the Δ in fasted FMD (C). The Δ in glucose iAUC_120min_ to the Δ in FMD iAUC_120min_ (D). The Δ in FMD iAUC_120min_ to the Δ in fasting respiratory exchange ratio (RER) (E) and the Δ in 60min RER (F). Open circles, LCD intervention; closed circles, LCD + INT intervention.

Low baseline fasting and iAUC_120min_ FMD was linked to enhanced fasting and iAUC_120min_ FMD post‐treatment (*r* = −0.71, *P* < 0.001; *r* = −0.89, *P* < 0.001, respectively). Weight loss correlated with increased FMD iAUC_120min_ (*r* = −0.40, *P* = 0.05). Increased fasting FMD was associated with increases in FFM (*r* = 0.40, *P* = 0.05; Fig. 2). Enhanced fitness was linked to increased fasting FMD (*r* = 0.43, *P* = 0.03; Fig. 2) and reduced FMD iAUC_120min_ (*r* = −0.44, *P* = 0.03; Fig. 2). This reduced FMD iAUC_120min_ was associated with decreased glucose iAUC_120min_ (*r* = 0.64, *P* = 0.001; Fig. 2) as well as increased fasting RER (*r* = −0.55, *P* = 0.004; Fig. 2) and 60min RER (*r* = −0.42, *P* = 0.04; Fig. 2). Lower glucose iAUC_120min_ was also associated with decreased ICAM‐1 (*r* = 0.47, *P* = 0.02) as well as increased fasting (*r* = −0.47, *P* = 0.02) and 60 min (*r* = −0.57, *P* = 0.004) RER.

## Discussion

Contrary to our hypothesis, 2 weeks of LCD or LCD + INT had no effect on fasted or postprandial (i.e., FMD iAUC_120min_) endothelial function as assessed by FMD in obese females. Interestingly, however, when examining women in the upper 50th percentile of responses, LCD + INT increased fasted FMD and decreased FMD iAUC_120min_ more than women undergoing LCD in the upper 50th percentile. These findings highlight that inter‐subject variation to a LCD with or without exercise confounded our ability to detect overall treatment effects. This variation in our study is not surprising as previous research has shown that ~25% of adult subjects do not demonstrate changes in fasted FMD after 8–18 weeks of exercise training (Green et al., 2014). Nevertheless, our findings build on previous short‐term (i.e., ≤2 weeks) research that showed a LCD improved fasted endothelial function in obese adults with hypertension (Sasaki et al., 2002) while INT exercise alone in postmenopausal women (Klonizakis et al., 2014) did not. We show that the combination of LCD + INT for 2 weeks may be more effective in some individuals at changing FMD when compared to an energy deficit matched LCD.

### Body composition and endothelial function

There are several reasons as to why INT would add to the effect of LCD on FMD. If individuals exercising lost more weight, then greater rises in fasting FMD would be expected (Joris et al., 2015). However, although we attempted to equate energy deficit between LCD and LCD + INT by refeeding 350kcal postexercise, those undergoing LCD had greater weight loss (~1kg) than LCD + INT. This suggests that weight loss per se is unlikely to explain the exercise effect, despite the observation that weight loss of ~2% was associated with increased FMD iAUC_120min_. In fact, work in obese adults with type 2 diabetes reported that a 30% reduction in calories or increased aerobic fitness with exercise training for 16 weeks did not raise FMD despite improved glycemic control and CVD risk factors (Wycherley et al., 2008). It is worth noting though that in our study, increases in fat‐free mass were also correlated with elevated fasted FMD. While not statistically significant, INT appeared to maintain lean mass compared to subtle reductions with LCD (*P* = 0.14). This may be physiologically relevant since reduced skeletal muscle mass is linked to decreased fasting FMD in overweight, older adults (Campos et al., 2017). Subsequently, exercise would seem to be an appropriate therapy to implement during a LCD to preserve typical losses in muscle mass seen with caloric restriction through, in part, protein synthesis (Tipton and Wolfe, 2001) and activation of vasodilator signals (e.g., nitric oxide) from endothelial cells (Nyberg et al., 2015). Collectively, the preservation or increase of skeletal muscle mass with 2 weeks of INT exercise combined with fat loss during LCD may be most beneficial for enhanced vascular health in obese females who respond to treatment.

### Aerobic fitness and endothelial function

Aerobic exercise training increases fasted brachial artery FMD in overweight and obese adult populations (Son et al., 2017). We build on these findings by showing that LCD + INT in the upper 50th percentile increased fasted FMD and decreased FMD iAUC_120min_ more than LCD in the upper 50th percentile. Why a rise in fasting FMD is paralleled by a decline in postprandial FMD is beyond the scope of this study, but it is consistent with literature showing that a glucose load reduces FMD in healthy individuals (Weiss et al., 2008). While it is often suggested that oxidative stress and inflammation promote this decline, we show that aerobic fitness induced adaptation of fasting FMD likely influenced the postprandial endothelial function response in LCD + INT compared to LCD (Franzoni et al., 2005; Kwon et al., 2011; Haykowsky et al., 2013). In fact, fitness is associated with increased nitric oxide bioavailability (Nosarev et al., 2015), arterial remodeling (Bloor, 2005), as well as decreased oxidative stress (Franzoni et al., 2005; Nosarev et al., 2015). As expected, we noted no change in arterial diameter following this short‐term intervention. This suggests that increases in FMD were due to more functional than structural change. One factor influencing function may relate to decreases in plasma glucose, that in turn, reduce oxidative stress and lower endothelial expression of ICAM‐1 (Ceriello et al., 1998). We show herein that a decrease in glucose iAUC_120min_ was in fact associated with a reduction in ICAM‐1. Since both interventions reduced ICAM‐1, our findings though suggest that fitness is unlikely to promote greater FMD change through solely an inflammatory mediated mechanism. Thus, future research is needed to understand the mechanism(s) by which different lifestyle therapies impact fasting and postprandial FMD given that CVD risk is associated more so with postprandial compared with fasting hyperglycemia (Lin et al., 2009).

### Postprandial endothelial function may relate to glucose utilization

Hyperinsulinemia is linked to lower fasted endothelial function through activation of MAPK induction of endothelian‐1 (Muniyappa et al., 2007). In the present study, LCD and LCD + INT similarly decreased hyperinsulinemia as assessed by insulin iAUC_120min_. This suggests that increased insulin sensitivity may contribute to FMD changes in these obese females. However, the reduction in hyperinsulinemia did not correlate with changes in fasted or postprandial FMD. Alternatively, we report that a decrease in circulating glucose was associated with not only decreases in FMD iAUC_120min_ but also increased fasted and 60 min CHO oxidation during the OGTT. This suggests that endothelial function changes are related to greater glucose utilization. We acknowledge while this later observation may seem counter‐intuitive at first, the reduction in FMD iAUC_120min_ may be due to the preservation of fasting FMD with exercise training (Das et al., 2018) or a re‐distribution of blood flow away from the brachial artery toward the microvasculature for increased delivery and use of glucose by skeletal muscle (Zheng and Liu, 2015). Indeed, it was recently demonstrated that resistance exercise training improved microvascular blood flow during an oral glucose load in obese adults with type 2 diabetes and this was independent of changes in postprandial FMD (Russell et al., 2017). Thus, further work in humans assessing microvasculature blood flow is required to confirm our hypothesis.

### Limitations

We did not measure non‐exercise physical activity and this could have contributed, in part, to changes in fasted and postprandial endothelial function as previous research has shown that alterations in physical activity patterns can influence FMD (Suboc et al., 2014). Nevertheless, VO_2_peak and lean body mass were not influenced by LCD suggesting that increases in non‐exercise physical activity were unlikely above that of LCD + INT. FMD was not corrected for shear stress as proposed by some (Thijssen et al., 2011; Olver et al., 2019), but not all (Welsch et al., 2002; Peretz et al., 2007; Jahn et al., 2016). Furthermore, we may have only captured peak and not maximal post‐ischemic diameter, since we did not continuously capture arterial diameter for 2‐min post‐deflation. Females in the present study were both pre‐ and postmenopausal. Postmenopausal females have a reduced fasting FMD compared to pre‐menopausal females (Santos‐Parker, 2017) due to decreases in ovarian function and estrogen levels (Moreau et al., 2013). However, we had a similar number of pre‐menopausal (*n* = 7 LCD, *n* = 5 LCD + INT) and postmenopausal (*n* = 5 LCD, *n* = 8 LCD + INT) females in each treatment group, and menopause status had no influence on fasted and iAUC_120min_ FMD (*P* ≥ 0.15) as determined by ANCOVA. We did not account for the phase of the menstrual cycle in our pre‐menopausal females because this was a 13‐day intervention. While we assume phase of the menstrual cycle would be distributed evenly among and between treatment groups by randomization, this warrants recognition as endothelial function is lowest in the early luteal phase and highest in the late follicular phase (Williams et al., 2005). Future short‐term work evaluating lifestyle therapy induced changes in FMD should account for the of phase of the menstrual cycle in pre‐menopausal females. A control group receiving no therapy as well as an exercise only treatment would have allowed for greater interpretation of the clinical relevance of endothelial function/dysfunction following lifestyle therapy on fasted and postprandial FMD. Lastly, caution should be used with interpretation of responders and nonresponders in this report. Despite some individuals being considered non‐responders because FMD did not increase, other cardiometabolic health benefits did occur. Moreover, we found that low baseline fasting and iAUC_120min_ FMD was linked to enhanced fasting and iAUC_120min_ FMD post‐treatment in obese females. This suggests that those people with lower fasting and/or postprandial FMD may have greater improvements post‐intervention. Together, these findings point toward the need to identify treatments that optimize precision medicine.

### Conclusion

Short‐term LCD alone or with INT had no effect on fasted or postprandial endothelial function in obese females. However, there was marked variation in response to LCD with or without exercise. In fact, this study showed that INT enhanced the effect of LCD, compared to LCD alone, on fasting FMD in obese females who responded and this was mirrored by low postprandial FMD stimulation. These changes in postprandial FMD were linked to aerobic fitness, glucose tolerance, and carbohydrate utilization, suggesting INT exercise may perhaps enhance nutrient delivery and utilization under caloric restriction conditions. Thus, INT with a LCD may be better for lowering type 2 diabetes and cardiovascular disease risk than LCD alone in obese females.

## Conflict of Interest

The authors declare no potential or perceived financial or other conflicts of interest.
